# Integrated Transcriptomic and Metabolomic Analysis of *G. hirsutum* and *G. barbadense* Responses to *Verticillium* Wilt Infection

**DOI:** 10.3390/ijms26010028

**Published:** 2024-12-24

**Authors:** Baoguang Xing, Pengtao Li, Yanfang Li, Bingkai Cui, Zhihao Sun, Yu Chen, Shaoliang Zhang, Qiankun Liu, Aiming Zhang, Liuan Hao, Xue Du, Xiaoyan Liu, Bei Wu, Renhai Peng, Shoulin Hu

**Affiliations:** 1 College of Agricultural, Tarim University, Alar 843300, China; 19937820150@163.com (B.X.); sybks10lyf25@163.com (Y.L.); cui20210521@163.com (B.C.); zsl001224@icloud.com (S.Z.); m904780569y@163.com (A.Z.); 2 School of Biotechnology and Food Engineering, Anyang Institute of Technology, Anyang 455000, China; lipengtao1056@126.com (P.L.); zhpaxl@163.com (Z.S.); cyu990324@163.com (Y.C.); liuthundering@163.com (Q.L.); hla19971016@163.com (L.H.); duxue101199@163.com (X.D.); ysjgd548@163.com (X.L.); 18734321011@163.com (B.W.)

**Keywords:** *Verticillium* wilt, transcriptome, metabolome, glutathione metabolism pathway

## Abstract

*Verticillium* wilt (VW) caused by *Verticillium dahliae* (Vd) is a devastating fungal cotton disease characterized by high pathogenicity, widespread distribution, and frequent variation. It leads to significant losses in both the yield and quality of cotton. Identifying key non-synonymous single nucleotide polymorphism (SNP) markers and crucial genes associated with VW resistance in *Gossypium hirsutum* and *Gossypium barbadense*, and subsequently breeding new disease-resistant varieties, are essential for VW management. Here, we sequenced the transcriptome and metabolome of roots of TM-1 (*G. hirsutum*) and Hai7124 (*G. barbadense*) after 0, 1, and 2 days of V991 inoculation. Transcriptome analysis identified a total of 72,752 genes, with 5814 differentially expressed genes (DEGs) determined through multiple group comparisons. KEGG enrichment analysis revealed that the key pathways enriched by DEGs obtained from both longitudinal and transverse comparisons contained the glutathione metabolism pathway. Metabolome analysis identified 995 metabolites, and 22 differentially accumulated metabolites (DAMs), which were correlated to pathways including glutathione metabolism, degradation of valine, leucine, and isoleucine, and biosynthesis of terpenoids, alkaloids, pyridine, and piperidine. The conjoint analysis of transcriptomic and metabolomic sequencing revealed DAMs and DEGs associated with the glutathione metabolism pathway, and the key candidate gene *GH_D11G2329* (glutathione S-transferase, *GSTF8*) potentially associated with cotton response to VW infection was selected. These findings establish a basis for investigating the mechanisms underlying the cotton plant’s resistance to VW.

## 1. Introduction

Cotton is a major source of renewable natural fibers for the textile industry and of vegetable oil for the food/feed industry [1,2]. Of the more than 50 *Gossypium* species identified thus far, four were domesticated for fiber production around the world, namely *G. herbaceum* (A1), *G. arboreum* (A2), *G. hirsutum* (AD1), *G. barbadense* (AD2) [3]. However, cotton growth and development were greatly affected by a range of biotic and abiotic stresses, such as saline-alkali soil, high temperatures, drought, pests, and pathogens, resulting in considerable yield losses and fiber quality reduction [4,5,6]. *Verticillium* wilt (VW), a soil-borne vascular disease that severely affects plants [7], is principally attributed to the pathogen *Verticillium dahliae* (Vd), which has a broad range of hosts, including more than 700 plant species in the Malvaceae, Fabaceae, Solanaceae, and Rosaceae families [8]. Once colonized in cotton plants, Vd mycelia or spores quickly spread through the xylem vessels via transpiration, obstructing the transport of water and nutrients in the vascular tissues. It also secreted toxic substances, eventually leading to symptoms of vascular bundle browning, leaf yellowing, wilting, defoliation, and even plant death [9,10]. Given the presence of Vd hyphae within plant vascular tissues and the dormancy of spores in the form of microsclerotia, conventional soil amendments, solar irradiation exposure, or fungicide treatments have been proved ineffective in VW disease control [4,11,12]. According to statistical data from the Agricultural Technology Extension Service Center of the Ministry of Agriculture and Rural Affairs of China in 2021, the losses caused by VW accounted for 32.94% of total cotton losses caused by various diseases in China [13]. In recent years, climate change, continuous monoculture, and frequent introduction of new cotton varieties and hybrids have exacerbated the occurrence of VW worldwide [14].

Despite being derived from the full hybridization between the same diploid ancestors, *G. hirsutum* and *G. barbadense* exhibit significant differences in plant traits such as fiber quality, yield, and environmental adaptability [15]. *G. hirsutum*, approximately accounting for 95% of global cotton production [16], is the most extensively cultivated species due to its advantages of high yield and strong adaptation, while it lacks resistant germplasm and superior fiber quality genes. *G. barbadense*, on the contrary, is renowned for its superior fiber length, strength, and ultra fineness, and VW resistance, contributing less than 5% to global cotton production [17]. Given these characteristics, it remains of great significance to introduce resistance genes from *G. barbadense* into *G*. *hirsutum* to significantly enhance its resistance to VW [18]. Therefore, investigating the distinctions between *G. hirsutum* and *G. barbadense* at the transcriptional and metabolic levels could help us understand the resistance mechanism of cotton to VW, identify key genes for VW resistance, and provide genetic materials for molecular breeding of VW resistance.

Transcriptomics is a discipline that studies the transcriptional levels in the cells or tissues of target organisms at specific developmental stages, locations, or physiological conditions, to dissect the molecular mechanisms behind complex biological pathways and trait regulatory networks [19,20]. Currently, transcriptome sequencing technology (RNA-seq) has been widely applied in plant disease resistance mechanisms to understand the interactions between plants and pathogens, and to identify relevant metabolic pathways and key genes [21]. Some transcriptomic results have identified metabolic pathways and differentially expressed genes (DEGs) associated with plant disease resistance; among these, hypersensitive response regulation and potassium ion transmembrane transport were highly activated in rice plant defense against blast disease [22], while chitinase, cytochrome *P450*, and *GST* were highly up-regulated in peanut defense against rust disease [23]. Similarity, we also noticed that the *GST* gene cluster was involved in cotton-resistant response against VW [8]. Metabolomics can detect changes in metabolites in cells or tissues, which helps determine the relationship between metabolites and plant biochemistry. Metabolome sequencing is also a key link connecting gene function and phenotypic variations, making it an important discipline after genomics, transcriptomics, and proteomics [24,25]. When pathogens invaded organs or tissues, plant metabolites were subjected to a series of changes, which might generate signaling molecules to activate the immune system to suppress or kill pathogens. In the metabolomic studies of *Solanaceae* bacterial wilt, 63 differentially accumulated metabolites (DAMs) were found to be closely related to the biosynthetic pathways of plant hormones, phenylpropanoids, and flavonoids [26]. In the metabolites of *Zanthoxylum bungeanum* Maxim rust disease, most of the metabolites were proved to belong to lipid and lipid-like molecules, phenylpropanoids, and polyketones, organic heterocyclic compounds [27]. In the interaction between susceptible/resistant watermelon varieties and *Fusarium oxysporum*, significant changes were observed in related metabolites, such as jasmonic acid-isoleucine, methyl jasmonate, melatonin, and lysine, implying their important roles in pathogen defense [28]. To understand the complex and variable biological processes, the conjoint analyses of RNA-seq and metabolomics could better reveal the molecular mechanisms of regulatory processes at different expression levels. Referring to the previous studies, it was observed through joint analysis that silver nanoparticles (AgNPs) activate plant hormone signaling and glutathione metabolism pathways, protecting rice seedlings from blast fungus infection [29]; it was also observed that HmF6′H1, an acetyl-coenzyme A 6′-hydroxylase, can promote the accumulation of simple coumarins in *Heracleum moellendorffii* Hance to resist infection by powdery mildew [30], and that DEGs and DAMs related to glutathione metabolism pathways can make great contributions to the response of rice to salt stress [31].

In this study, VW-susceptible TM-1 and VW-resistant Hai7124 were selected to perform transcriptome and metabonomic analyses. Root samples were separately collected at 0, 1, and 2 days after V991 inoculation for sequencing analysis. Multiple pair comparisons among the samples generated a number of DEGs and DAMs, which were then submitted to enrichment analysis in Kyoto Encyclopedia of Genes and Genomes (KEGG) pathways. Combining identified key candidate genes and metabolites, this study lays the foundation for the functional validation of candidate genes in subsequent research.

## 2. Results

### 2.1. Sequencing of the Transcriptome and Analysis of Quality Control

To systematically elucidate the critical genes and signaling pathways in plant defense mechanisms against VW infection, 18 RNA-seq libraries were constructed from root samples of the representative varieties TM-1 and Hai7124. The samples were taken at 0, 1, and 2 days after inoculation (DAI) of V991. After removing low-quality reads, a total of 802.88 million clean reads were obtained, with an average clean read (M) of 38.12–49.15 per sample. The Q30 values of all the samples exceeded 91.95%, and the GC content ranged from 43.43% to 45.61%. Mapping the clean reads of each sample to the reference genome showed alignment efficiencies ranging from 94.04% to 97.27% (Table S1).

**Figure 1:**
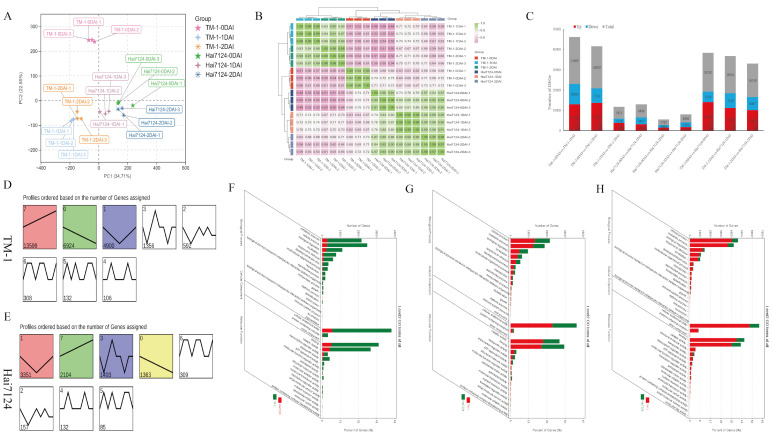
Analysis of Expression Patterns and Identification of Genes with Differential Expression (**A**) PCA analysis of the 18 samples. (**B**) Correlation clustering heatmap of the 18 samples. (**C**) DEGs in the 9 pairwise comparisons. (**D**) Trend analysis results of DEGs in TM-1. (**E**) Trend analysis results of DEGs in Hai7124. (**F**) GO enrichment results of DEGs in expression profile 0 for TM-1 and Hai7124. (**G**) GO enrichment results of DEGs in expression profile 1 for TM-1 and Hai7124. (**H**) GO enrichment results of DEGs in expression profile 7 for TM-1 and Hai7124. Totally, 1692 DEGs were identified in the comparison between different infection stages of TM-1 at 0, 1, and 2 days after V991 inoculation (Figure 2A). KEGG enrichment analysis of these DEGs showed that the enriched pathways included ribosome, terpenoid biosynthesis, plant hormone signal transduction, circadian rhythm-plant and glutathione metabolism (Figure 2B). Similarly, 293 DEGs were identified in the comparison between different infection stages of Hai7124 at 0, 1, and 2 days after V991 inoculation (Figure 2C). KEGG enrichment analysis of these DEGs showed that the enriched pathways included biosynthesis of secondary metabolites, terpenoid biosynthesis, isoflavonoid biosynthesis, carotenoid biosynthesis, plant–pathogen interactions, and glutathione metabolism (Figure 2D). The comparison between the two varieties at the same time point after V991 inoculation resulted in 5814 DEGs (Figure 2E), and the enriched KEGG pathways included biosynthesis of secondary metabolites, glutathione metabolism, sesquiterpenoid and triterpenoid/terpenoid compound biosynthesis, and isoflavonoid biosynthesis (Figure 2F). The metabolism of glutathione was enriched in both comparisons of DEGs between time points and between varieties at same time point, suggesting its potentially important roles in cotton resistance against Vd invasion.

Based on the annotated reference genome, a total of 72,752 expressed genes were identified in our RNA-Seq dataset. The expression levels of these genes were quantified using the number of fragments per kilobase of transcript per million mapped reads (FPKM). To confirm the accuracy of the RNA-seq data, principal component analysis (PCA) across the 18 samples was performed, and two main components, namely PC1 and PC2, were obtained, which contributed 34.71% and 22.85% of the total variation, respectively. The three biological replicates at different time points showed a high similarity between different samples (Figure 1A). Pearson’s correlation coefficient (PCC) analysis on all the RNA-seq samples showed a gene expression correlation of over 90% for each sample (Figure 1B). These results suggest that the transcriptome data are of high quality which meets the standards for the subsequent DEG identification and their functional enrichment analysis.

### 2.2. DEG Identification and Expression Pattern Analysis

Nine pairwise comparisons were performed on all the samples, resulting in 39,976 DEGs (Figure 1C). By comparing the different infection stages within the susceptible TM-1 and within the resistant Hai7124, there were more DEGs identified in the former (TM-1-0DAI vs. TM-1-1DAI, TM-1-0DAI vs. TM-1-2DAI, and TM-1-1DAI vs. TM-1-2DAI) than in the latter (Hai7124-0DAI vs. Hai7124-1DAI, Hai7124-0DAI vs. Hai7124-2DAI, and Hai7124-1DAI vs. Hai7124-2DAI). A similar phenomenon was also observed during early infection (0DAI to 1DAI). The difference between TM-1 and Hai7124 was also manifested in the period when the minimum number of DEGs appears, with the former showing the smallest DEGs in the comparison of TM-1-1DAI vs. TM-1-2DAI, while the latter showed Hai7124-0DAI vs. Hai7124-2DAI. For the pairwise comparisons of different varieties at the same infection stage (DAI), it was observed that the pair TM-1-0DAI vs. Hai7124-0DAI had the highest number of DEGs, while the pair TM-1-2DAI vs. Hai7124-2DAI had the lowest number of DEGs. In addition, more up-regulated DEGs were identified in the resistant variety than in susceptible varieties.

To investigate the spatiotemporal expression patterns of genes, 28,069 DEGs in TM-1 and 8904 genes in Hai7124 were separately submitted to short time-series expression miner (STEM) analysis (Figure 1D,E). The findings indicated that a substantial majority of the DEGs in TM-1, 90.57% of the total 28,069 DEGs, were significantly clustered into 3 distinct expression profiles. Among these profiles, in profile 7, which clustered 13,599 DEGs, 48.44%, showed a continuous up-regulation trend; in profile 0, which clustered 6924 DEGs, 24.67%, showed an overall down-regulation trend; and in profile 1, which clustered 4900 DEGs, 17.46%, showed an initial down-regulation followed by an up-regulation trend. On the other hand, 8221 DEGs (92.33% of the total) in Hai7124 were clustered in 4 expression profiles. Among these profiles, profile 1, which clustered3,351 DEGs, 37.63%, showed an initial down-regulation followed by sustained up-regulation trend; profile 7, which clustered 2104 DEGs, 23.63%, showed a continuous up-regulation trend; profile 0, which clustered 1363 DEGs, 15.31%, demonstrated a sustained down-regulation trend; and profile 3, which clustered 1403 DEGs, 15.76%, showed a periodic pattern of initial down-regulation, followed by up-regulation, and then another down-regulation.

### 2.3. Functional Enrichment Analysis of DEGs

The functional enrichment analyses were performed on the DEGs in the significantly clustered expression profiles (0, 1, and 7) in TM-1 and Hai7124 using the GO and KEGG databases. In profile 0, the DEGs were enriched in metabolic processes, cellular processes, cellular anatomical entities, and catalytic activities in the first three GO terms of biological processes (BP), cellular components (CC), and molecular functions (MF) (Figure 1F). As profile 0 exhibited a sustained down-regulation trend, which was more prevalent in susceptible TM-1 than in resistant Hai7124, KEGG enrichment analysis showed that the common pathways in both TM-1 and Hai7124 included hormone signal transduction, circadian rhythm-plant, MAPK signaling pathway-plant, and phosphatidylinositol signaling system (Figure S1A).

In profile 1, the DEGs were enriched in the GO terms of processes related to cellular processes, metabolic processes, cellular anatomical entities, catalytic activities, and binding (Figure 1G). KEGG enrichment analysis revealed that the common pathways in both genotypes included flavonoid biosynthesis, circadian rhythm-plant, biosynthesis of various plant secondary metabolites, and plant hormone signal transduction (Figure S1B). In particular, the significant enrichment of profile 1 DEGs in the biosynthesis of secondary metabolites and plant hormone signal transduction suggested their crucial role in resisting pathogen invasion.

In profile 7, the DEGs were enriched in the GO terms of cellular processes, metabolic processes, cellular anatomical entities, binding, and catalytic activities (Figure 1H). KEGG enrichment analysis revealed that the key pathways in TM-1 were related to ribosome biogenesis, carotenoid biosynthesis, base excision repair, nitrogen metabolism, and endocytosis, which are associated with growth and development. In contrast, the key pathways in Hai7124 were associated with plant defense, such as ABC transporters, phenylpropanoid biosynthesis, flavonoid biosynthesis, and plant–pathogen interactions (Figure S1C).

### 2.4. Metabolomic Comparative Analysis of Different Strains

**Figure 3:**
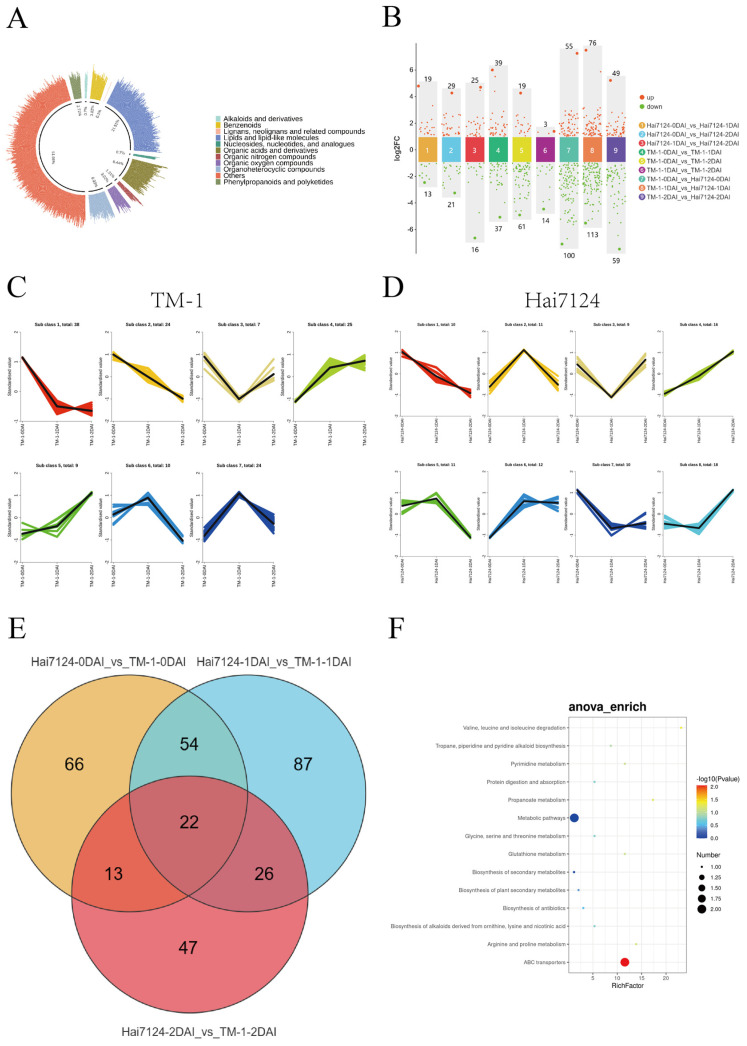
Comparative Metabolomics Analysis of Different Strains (**A**) Secondary classification of 995 metabolites. (**B**) Scatter plots of differential analysis across 9 groups. (**C**) Clustering analysis results of DAMs in TM-1. (**D**) Clustering analysis results of DAMs in Hai7124. (**E**) Venn diagram of 22 common DAMs at the same infection stage across different materials. (**F**) Enrichment analysis results of in common DAMs at the same infection stage across different materials.

To investigate the metabolic differences between TM-1 and Hai7124 during cotton VW infection, untargeted metabolomic sequencing was performed on the 18 samples, and quality control analysis was performed on the sequencing data. PCA analysis showed that PC1 scored 20.81% and PC2 scored 13.81%, indicating significant metabolic phenotype differences between the two samples, and high similarity among the three biological replicates (Figure S2A). The correlation coefficients exceeding 0.8 in the correlation analysis of the QC samples suggested significant consistency (Figure S2B). A total of 995 metabolites were identified in the 18 samples, which were categorized into 11 major classes: alkaloids and derivatives (7, 0.7%); benzenoids (38, 3.82%); lignans, neolignans, and related compounds (1, 0.1%); lipids and lipid-like molecules (215, 21.51%); nucleosides, nucleotides, and analogues (7, 0.7%); organic acids and derivatives (84, 8.44%); organic nitrogen compounds (13, 1.31%); organic oxygen compounds (30, 3.02%;, organoheterocyclic compounds (60, 6.03%); others (514, 51.66%); and phenylpropanoids and polyketides (27, 2.71%) (Figure 3A).

Based on the general threshold criteria of Variable Importance in Projection (VIP) equal to or greater than 1, Fold Change Screening Value equals to 2, and *p*-value less than 0.05, a total of 402 DAMs were identified through nine groups of pairwise comparisons. In the comparisons between different time points after V991 inoculation in the same variety, it was found that the largest numbers of DAMs were identified in the groups of TM-1-0DAI vs. TM-1-2DAI (80 DAMs) and Hai7124-1DAI vs. Hai7124-2DAI (50 DAMs), while the least numbers of DAMs were observed in TM-1-1DAI vs. TM-1-2DAI and Hai7124-0DAI vs. Hai7124-1DAI (Figure 3B). Significantly, different varieties showed DAMs at the same infecting time points; the maximum and minimum DAMs were identified in the groups of TM-1-1DAI vs. Hai7124-1DAI (189 ones) and TM-1-2DAI vs. Hai7124-2DAI (108 ones), respectively. After removing duplicates, a total of 137 and 97 DAMs were separately obtained in TM-1 and Hai7124 via different infection time point comparisons. Subsequently, via K-means clustering analysis, the DAMs of TM-1 and Hai7124, were classified into 7 and 8 clusters, respectively (Figure 3C,D). In TM-1, subclusters 4 and 5 demonstrated a consistent increasing expression trend with time advances after V991 infection. Conversely, subclusters 1 and 2 showed a decreasing expression trend with time advances after infection. In Hai7124, subcluster 4 displayed a consistent increasing expression trend with time advances after infection. Subcluster 1 showed a decreasing expression trend with time advances. In both TM-1 and Hai7124, carboxylic acid derivatives and sphingolipids showed a decreasing expression trend with time advances after V991 infection V991, while glycerophospholipids and fatty acyls exhibited a continuous increasing expression trend (Table S2). It could be speculated that glycerophospholipids and fatty acyls play important roles in early resistant responses against Vd invasion. Comparison of DAMs between TM-1 and Hai7124 at the identical time point identified 22 common DAMs (Figure 3E). KEGG enrichment analysis indicated that the majority of common DAMs were significant enriched in pathways of valine, leucine, and isoleucine degradation, glutathione metabolism, metabolic pathways, and propanoate metabolism (Figure 3F).

### 2.5. Conjoint Analysis of DEGs and DAMs

**Figure 4:**
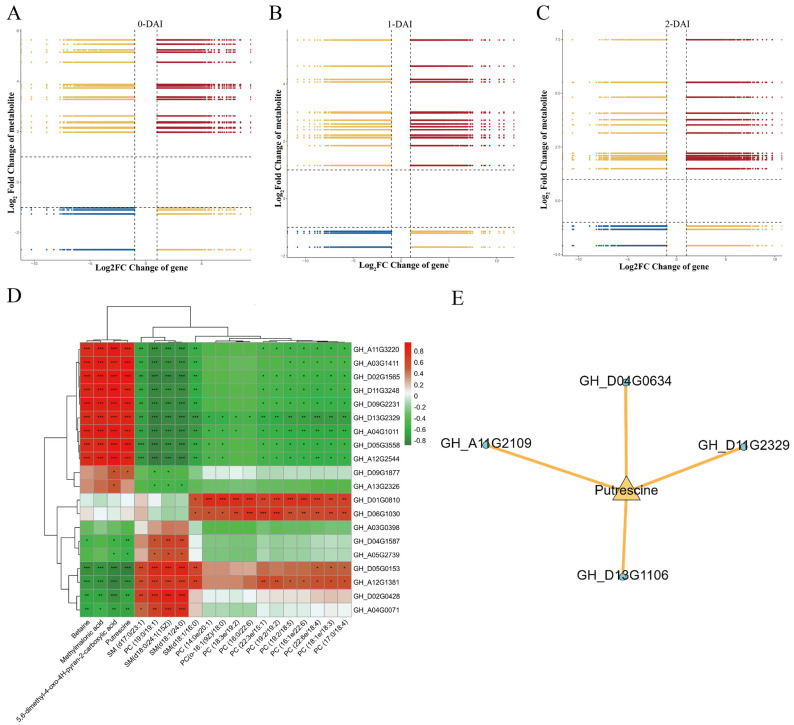
Conjoint analysis of DEGs and DAMs (**A**) Nine-quadrant plot of DEGs and DAMs in response to V991 infection in cotton at day 0 (In the third quadrant, the expression patterns of DEGs and DAMs are consistent and up-regulated at the same time. In the seventh quadrant, the expression patterns of DEGs and DAMs are consistent and down-regulated simultaneously. In the first and ninth quadrants, the expression patterns of DEGs and DAMs are opposite, one up-regulated and the other down-regulated. (**B**,**C**) comments are the same). (**B**) Nine-quadrant plot of DEGs and DAMs in response to V991 infection in cotton at day 1. (**C**) Nine-quadrant plot of DEGs and DAMs in response to V991 infection in cotton at day 2. (**D**) Correlation clustering results of DEGs and DAMs in response to V991 infection in cotton(Use *p*-values to indicate significant differences. * means the significant level is less than 0.05, ** means the significant level is less than 0.01, and *** means the significant level is less than 0.001). (**E**) Network diagram of annotated DEGs involved in glutathione metabolism.

To dissect the macroscopic developmental processes of biological systems, a joint analysis of transcriptomic and metabolomic data of the two cultivars was performed in this study. The results revealed that the pathways that DAMs and DEGs co-enriched included glutathione metabolism, valine, leucine, and isoleucine degradation, propionate metabolism, ABC transport, secondary metabolite biosynthesis, and metabolic pathways (Figure S3). Further calculation of Pearson correlation coefficients was performed for the 5814 DEGs and 22 DAMs, generating nine quadrant plots (Figure 4A–C) and a correlation clustering heatmap (Figure 4D). The expression patterns of DEGs and DAMs in the first and ninth quadrants were opposite, implying that the changes in these DAMs might be negatively regulated by DEGs. In the third and seventh quadrants, the expression patterns of DEGs and DAMs showed consistency, implying that the variations of these DAMs might be positively regulated by the corresponding DEGs. In addition, during the processes of V991 infection in TM-1 and Hai7124 (0, 1, and 2 days), the metabolite putrescine was positively regulated by *GH_D04G0634*, *GH_D11G2329*, and *GH_D13G1106*. The correlation clustering heatmap results showed that the red part represents a positive correlation between DEGs and DAMs, while the green part represents a negative correlation. Notably, putrescine was significantly positively correlated with *GH_D11G2329*.

**Figure 5:**
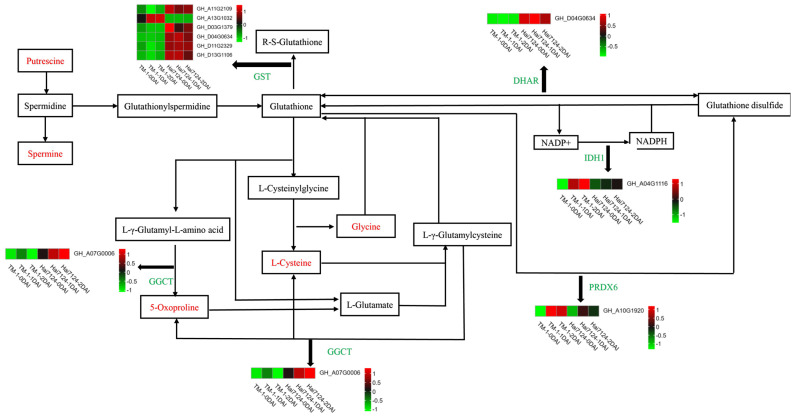
Signaling Pathway of Glutathione Metabolism and Heatmap of Selected Annotated DEGs and DAMs.

Based on the joint analysis of DEGs and DAMs, it could be speculated that the glutathione metabolism pathway is vital for resisting Vd invasion. The correlation analysis revealed a significant association between putrescine and corresponding DEGs, such as the gene-encoding glutathione S-transferase (GST). Network analysis demonstrated that DEGs and DAMs in the glutathione metabolism signaling pathway formed hub genes in the network, supporting the aforementioned correlation analysis (Figure 4E and Table S3). Additionally, a schematic diagram of the glutathione metabolism pathway was constructed (Figure 5). In total, 9 DEGs were screened in TM-1 and Hai7124 at 0, 1, and 2 DAI through transcriptomic analysis, which belonged to GST; glutathione dehydrogenase/transferase (DHAR); peroxiredoxin 6 (PRDX6); isocitrate dehydrogenase 1 (IDH1); and γ-glutamylcyclotransferase (GGCT). Meanwhile, 5 DAMs involved in this pathway were identified by metabolomic analysis, namely putrescine, spermine, glycine, L-cysteine, and 5-oxoproline.

In the findings from the joint analysis of transcriptomics and metabolomics, the DEG *GH_D11G2329* (*GHGSTF8*) participating in the glutathione metabolism pathway showed an up-regulated expression pattern in TM-1 during V991 infection, indicating a potential role in VW resistance.

### 2.6. Verification of Gene Expression qRT-PCR

**Figure 6:**
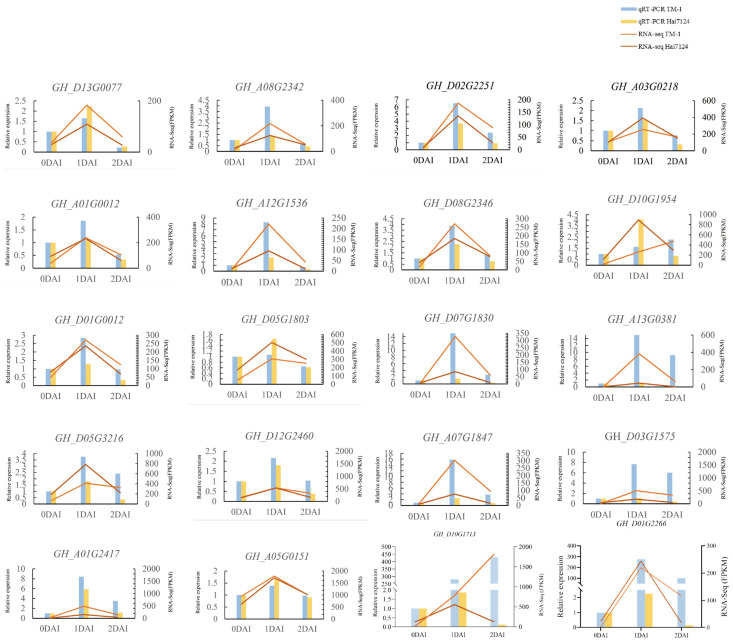
Results from qRT-PCR Randomly Chosen Genes.

To validate the accuracy of the transcriptomic analysis results, 20 genes were randomly selected and analyzed using quantitative real-time polymerase chain reaction (qRT-PCR) experiments (Figure 6). Detailed information on the primer design is listed in Table S4. The housekeeping gene *GhUBQ7* (DQ116441) served as a reference control for the analysis of relative quantification. The results confirmed that the expression trends observed in qRT-PCR were consistent with the transcriptomic sequencing results, indicating the reliability of the transcriptomic sequencing.

## 3. Discussion

Cotton, as an important economic crop, makes great contributions to global economic development. Currently, more than 50 species have been identified within the cotton genus, among which *G. hirsutum* and *G. barbadense* are widely cultivated allotetraploid species [32]. *G. hirsutum* has the traits of high yields yet average fiber quality, while *G. barbadense* exhibits the opposite characteristics [33]. However, the VW disease could cause annual yield losses of approximately 10–35%, since the majority of *G. hirsutum* varieties are susceptible to VW, ultimately posing a significant economic threat worldwide [34]. Conventional control measures such as crop rotation or chemical fumigation have been proved ineffective against VW, due to fact that the pathogen survives in the environment in the form of hyphae, spores, and microsclerotia. It is stimulated by cotton root exudates and begins to initiate infection at the root tips. During the infection process, the hyphae penetrate the root epidermal cells, move through the xylem, and colonize the vascular tissues, eventually reaching the petiole base. This can lead to browning of the cotton vascular bundles, leaf yellowing, wilting, defoliation, and even plant death [6,35]. Moreover, the pathogens exist in the soil in the form of dormant spores, enabling them to infect multiple generations of crops [36]. Therefore, it is necessary for breeders to focus on developing resistant varieties to meet production needs. However, the condition we faced is that *G. hirsutum* varieties lack resistance genes and quantitative trait loci (QTL) associated with VW [2]. To address this issue, introducing genes of VW resistance from *G. barbadense* into *G. hirsutum* provides a useful strategy to significantly enhance cotton defense [18]. Meanwhile, key SNP loci related to VW resistance in *G. hirsutum* and *G. barbadense* could be used to identify resistance genes and breed resistant varieties. For instance, critical SNP substitutions in *GhWAKL14* (A/C) resulted in amino acid changes (S/R), enhancing resistance of *G*. *hirsutum* but rendering *G. barbadense* susceptible to *Fusarium wilt* [37]. Similarly, critical SNP substitutions in *GhGLR4.8* (C/A) led to amino acid alterations (L/I), enhancing *Fusarium wilt* resistance in *G. hirsutum* [38]. Similar methods have also been applied to the defense mechanisms of economically important crops against pathogens, such as onions [39], wheat [40], soybeans [41], and citrus [42].

### 3.1. RNA-Seq of Cotton in Response to VW

Currently, RNA-seq has been widely applied to study the interaction mechanisms between plants and pathogens, such as in maize [43], wheat [44], and rice [45]. In this study, RNA-seq analysis was performed on the roots of TM-1 and Hai7124 at 0, 1, and 2 DPAs after VW infection. Pairwise analysis between different samples identified a total of 39,976 DEGs. Trend analysis of temporal expression patterns of TM-1 and Hai7124 DEGs revealed three common expression patterns. Expression profile 0 showed a continuous downregulation trend, which was more prevalent in the susceptible TM-1 than in the resistant Hai7124. Expression profile 7 exhibited a continuous upregulation trend, with TM-1 showing more key pathways related to growth and development, including ribosome replication, base excision repair, nitrogen metabolism, and endocytosis. In contrast, Hai7124 showed more active pathways related to plant defense, including secondary metabolite biosynthesis, phenylpropane biosynthesis, flavonoid biosynthesis, and plant–pathogen interactions. The findings indicate that Hai7124 may possess the key genes conferring resistance to VW. Research indicates that during VW infection, cotton notably increases the accumulation of secondary metabolites [46]. The phenylpropanoid metabolic pathway produces numerous natural compounds, such as flavonoids and lignin, which are extensively involved in various physiological activities in plants [47]. In cotton, the silencing of the *GhCOMT* gene results in reduced lignin content and decreased resistance to Vd [48]. Conversely, overexpression of *GhLac1* leads to increased lignin content and enhanced resistance to Vd [49]. Flavonoid biosynthesis is crucial for muskmelon defense against downy mildew [50]. KEGG-enriched pathways via longitudinal and transverse comparisons of TM-1 and Hai7124 DEGs included the glutathione metabolism pathway. Studies indicated that glutathione metabolism is crucial for antioxidant stress and pathogenicity within *Fusarium graminearum* [51], cadmium tolerance in *Arabidopsis* [52], and early resistance to *Bacterial wilt* in tobacco [53]. This suggested that our RNA-seq results highly align with these findings, indicating them to be crucial for combating VW infection.

### 3.2. Metabolomic Analysis of Cotton in Response to VW

Currently, metabolomics has been primarily applied to analyze the types, quantities, intrinsic factors, and variations in plant metabolites under different environmental conditions, making it a valuable tool for studying plant–pathogen interactions [54]. In this study, we identified altogether 402 DAMs through pairwise comparisons. K-means clustering analysis revealed that the expression levels of glycerophospholipids and fatty acyls in TM-1 and Hai7124 increased continuously after the Vd infection. Previous studies have indicated that the accumulation of glycerophospholipids is vital for barley under salt stress [55]; compounds composed of flavonoids, amino acids, and glycerophospholipids in Tibetan hulless barley respond to osmotic stress (cold, high salt, and drought) [56]; metabolites such as fatty acyls are significantly induced in salt-tolerant wild rice compared to salt-sensitive rice [57]. Therefore, it is speculated that glycerophospholipids and fatty acyls play key roles in early resistance to VW invasion. In the longitudinal comparison analysis of TM-1 and Hai7124 infected with V991, a total of 22 DAMs were identified. KEGG analysis of these 22 DAMs indicated a significant enrichment in the glutathione metabolism pathway. Currently, research on cotton VW has mainly focused on gene expression levels, with fewer studies on the metabolite level. The findings of this study complement this aspect and establish a basis for cotton resistance to VW. Metabolomic analysis has been widely applied in other plant disease resistance studies. For example, under *Phytophthora infestans* stress, resistant potato varieties accumulate salicylic acid, phenylpropanoids, terpenoids, and arachidonic acid, which is vital for thickening the cell wall [58]. In multiomics studies, metabolites such as linolenic acid, vitamin E, choline, and riboflavin contribute to enhancing resistance to rice blast disease [59]. In *Fusarium wilt* disease research in wheat, the accumulation of proline (Pro) and alanine (Ala) enhances wheat resistance, whereas a decrease in cysteine (Cys) exacerbates sensitivity [60]. After infection with tomato yellow leaf curl virus, significant changes in the abundance of polyamines, phenols, and indoles were detected in resistant and susceptible tomato varieties. Salicylic acid accumulated significantly in resistant tomatoes, suggesting its important role in tomato disease resistance [61].

### 3.3. Conjoint Analysis of Metabolome and Transcriptome in Cotton Response to VW

When pathogens invade plants, they trigger the expression of genes associated with plant defense mechanisms, thereby synthesizing disease-resistant proteins or other compounds that resist pathogen invasion. Therefore, the combined analysis of transcriptome and metabolome is utilized to screen target genes and to elucidate target metabolic pathways [62]. For example, the enzymes encoded by the tau cluster of the GST family may play a crucial role in maintaining the delicate balance between the production and scavenging of H_2_O_2_. This establishes a new equilibrium that enhances the functions of CAT and POD, thereby strengthening the plant’s response to Vd [8]. This study identified nine enriched pathways in cotton response to VW through combined KEGG analysis of transcriptome and metabolome. The longitudinal and transverse pairwise analysis of DEGs in the transcriptome and the longitudinal comparison analysis of DAMs in the metabolome collectively enriched the glutathione metabolism pathway. The results of the nine-quadrant map and correlation clustering heatmap indicated that the metabolite spermine in the glutathione metabolism pathway was positively regulated by the annotated DEGs in this pathway. Furthermore, the construction of the glutathione metabolism pathway diagram revealed the involvement of spermine, spermidine, glutamic acid, L-cysteine, and 5-oxoproline in this pathway. Exogenous application of polyamines (spermine, spermidine, and spermine) significantly increased the activities of superoxide dismutase (SOD), catalase (CAT), and glutathione reductase (GR), maintaining their internal homeostasis of apricot and enhancing its resistance to black spot disease [63]. Glycine can increase soil absorption of heavy metals, alleviating plant toxicity caused by heavy metals [64]. Hydrogen sulfide and cysteine can alleviate cadmium-induced growth inhibition in Arabidopsis and enhance its tolerance to cadmium [65]. In the study of necrotic pathogens, it was found that the infected alfalfa significantly accumulated 5-oxoproline and malic acid, possibly enhancing its resistance to necrotic pathogens [66]. RNA-seq identified GST, DHAR, PRDX6, IDH1, and GGCT as participating in this pathway, with correlation analysis showing a significant association between putrescine and the gene-encoding GST. In this study, the DEG *GH_D11G2329* (*GSTF8*) was selected from the glutathione metabolism pathway. GSTs are a multifunctional enzyme encoded by a multigene superfamily that plays a detoxification role in various plants [67]. In the study of salt tolerance in the rice landrace HD961, a comprehensive analysis of the transcriptome and metabolome revealed that the glutathione metabolism pathway was significantly enriched, and a total of seven metabolites and 48 DEGs identified, most of which belong to the GST family. Among these, L-cysteine was the most active compound in response to salt stress. *GSTU2* showed a significant correlation with metabolites such as L-cysteine, spermidine, and L-ornithine. Additionally, there was a notable correlation between GSH and 16 genes. These findings suggest that these specific genes and glutathione metabolites play a crucial role in rice’s response to salt stress [31]. Previous research has emphasized the vital importance of GSTs in combating peanut rust [23], cotton VW [8], and wheat *Powdery mildew* [68]. SA (salicylic acid) is one of the primary defense-related hormones involved in the response to various biotrophic and hemibiotrophic pathogens, and plays an auxiliary role against necrotrophic pathogens [69]. Vd initially behaves as a biotrophic pathogen during the early stages of infection but shifts to a necrotrophic lifestyle in the later stages. Therefore, the SA signaling pathway is required to confer resistance to Vd [13]. Additionally, the complex metabolic pathways of plant secondary metabolites often intersect with SA signaling. In the glutathione metabolism pathway, *GaGSTF9* may regulate reactive oxygen species content via catalyzing the reduction in tripeptide glutathione (GSH), thereby positively influencing cotton resistance to Vd and SA content [70]. Additionally, the constitutive overexpression of the gene encoding the key rate-limiting enzyme in spermine biosynthesis, *GhSAMDC* (S-adenosylmethionine decarboxylase), in *Arabidopsis thaliana* enhances resistance to Vd by activating SA signaling pathways [71]. Therefore, it is believed that the glutathione metabolism pathway plays an important role in early defense mechanisms of cotton against VW infection. In consideration of the relative disadvantages of traditional hybridization, self-cross, and backcross techniques adopted between TM-1 and Hai7124, such as long period, large population, and heavy workload, the candidate genes are even suggested to perform the genetic transformation for further cotton breeding. The more precise and efficient methods, including transgenic modification and gene editing by CRISPR-cas9 system, could break the reproductive isolation and trait linkage, finally achieving synchronous improvement in fiber yield, quality, and multiple resistances.

## 4. Materials and Methods

### 4.1. Cultivation of Materials and Preparation of Strains

The cotton varieties TM-1 (*G. hirsutum*) and Hai7124 (*G. barbadense*) were preserved in our laboratory (TM-1 and Hai7124 were provided by Dr. Fang Liu’s research group at the Institute of Cotton Research). TM-1 and Hai7124 are two important cultivated cotton varieties. TM-1 exhibits stable agronomic traits and medium-level fiber quality, and it does not contain Bt, making it an excellent recipient for the introduction of exogenous genes. It is frequently used in constructing genetic linkage maps, serving as background material for developing substitution lines [72]. Hai7124 is characterized by its ultra-long or superfine quality fibers and exhibits strong resistance to VW [17]. The VW pathogen strain used was the highly pathogenic isolate V991 of Vd Kleb (V991 was provided by Professor Yingfan Cai from Henan University). The seeds of both cotton varieties were defuzzed using concentrated sulfuric acid, thoroughly washed with water, and air-dried. Uniform and fully developed seeds were selected and planted in a sterilized substrate composed of sand and vermiculite in a ratio of 3:2 in the greenhouse of Anyang Institute of Technology. The cultivation took place within a controlled greenhouse environment maintained at a temperature of 28 °C, adhering to a photoperiod of 16 h of light followed by 8 h of darkness, maintaining a relative humidity of 68%. Once the first true leaves of the cotton plants fully expanded, root tissues with consistent growth were collected as the wild-type (WT) control treatment. These roots were quickly transferred into collection tube that had been pre-cooled in liquid nitrogen to ensure the preservation of the samples. They were subsequently cryopreserved in liquid nitrogen and stored in an ultra-low temperature freezer set at −80 °C for future use. Additionally, we selected cotton plants exhibiting consistent growth for infection using the V991 strain (2 mL of V991 per plant), and root tissues were collected at days 1 and 2 post-infection. We rinsed the roots with tap water to remove soil, and then we collected the primary roots of the cotton plants below the hypocotyl. We quickly wrapped the root in aluminum foil and placed it into a pre-cooled collection tube with liquid nitrogen (0.5 g per replicate, consistent with the collection method for WT). One portion of the root tissues was allocated for transcriptome sequencing, while another portion was designated for metabolome sequencing.

The preserved pathogen strain was cultured on potato dextrose agar (PDA) plates. These plates were placed in an inverted position in a dark incubator with temperature controlled at 25 °C for 7 days. Subsequently, approximately 2 mm^2^ fungal plaques were transferred to Czapek Dox liquid medium using sterilized toothpicks and placed at 25 °C in the dark incubator with a shaking speed of 150 r/min for 7 days. Prior to inoculation, the mycelium was filtered through four layers of gauze. The density of the resulting spore suspension was then measured using a hemocytometer. Then the suspension was diluted with ddH_2_O to a concentration of 1 × 10^7^ spores/mL, and the cotton roots were inoculated [73].

### 4.2. Extraction of RNA and Construction of Library

The FastPure Universal Plant Total RNA Isolation Kit (RC411) from Vazyme was used to isolate the total RNA. The RNA’s concentration and quality were assessed using a NanoDrop 2000 spectrophotometer, and agarose gel electrophoresis was performed to check for RNA contamination and degradation. Samples that passed the quality standards were forwarded to Igenebook Biotechnology Co., Ltd., Wuhan, China for library construction and sequencing.

The library construction used the NEBNext^®^ Ultra™ RNA Library Prep Kit (NEB, Ipswich, MA, USA), and PCR amplification was performed to obtain cDNA libraries. A Qubit 4.0 Fluorometer was employed to measure the concentration of the libraries, while the fragment sizes were assessed with a QSep400. The Qubit Fluorometer was again used to quantify the libraries. Once the libraries passed quality control, 18 cDNA libraries (from two cotton strains at 0, 1, and 2 days) were sequenced using the combinatorial probe-anchor synthesis (cPAS) technology.

### 4.3. Transcriptome Sequencing

The initial sequencing data were processed to filter out impurities to obtain clean reads. The reference genome of *Gossypium hirsutum* was downloaded, and alignment was performed using HISAT2 (https://www.cottongen.org/species/Gossypium_hirsutum/UTX-TM1_v2.1, accessed on 13 March 2024). The clean reads were assembled and expression levels were evaluated using StringTie version 2.0.4 software. RNA differential expression analysis between two different samples was performed by DESeq2 software (https://www.omicshare.com/tools/, accessed on 21 August 2024) (and by the edgeR package in R version 4.4.1 for comparisons between two samples). The genes/transcripts identified based on a false discovery rate (FDR) < 0.05 and absolute fold change ≥ 2 were considered differentially expressed genes/transcripts. OmicShare was employed to conduct functional enrichment analysis for KEGG pathways and GO categories (https://www.omicshare.com/tools/, accessed on 23 August 2024).

### 4.4. Metabolic Analysis (LC−MS/MS)

Root tissue samples were taken at 0, 1, and 2 DAI after being inoculated with V991 and subsequently sent to Igenebook Biotechnology Co., Ltd. for untargeted metabolite profiling. The extraction and analysis of the metabolites were performed through liquid chromatography/mass spectrometry (LC-MS) [74]. The raw data files were loaded into TraceFinder version 3.2.0 for analysis. Each metabolite was screened according to criteria including retention time and mass-to-charge ratio. To ensure accurate identification of metabolites, peak alignment was conducted on various samples. During the alignment process, we maintained a retention time deviation of 0.2 min and allowed for a mass deviation of 5 parts per million (ppm) to ensure accurate identification. Peak areas were quantified, and spectral matching was conducted against mzCloud alongside a locally established database (https://www.mzcloud.org/, accessed on 30 January 2024). In order to ensure accuracy in the data, the raw quantitative results were subjected to normalization processes. This step was crucial for facilitating the identification and relative quantification of metabolites present in the samples under investigation. The metabolites that were identified during this process were meticulously annotated with reference to several authoritative databases. Specifically, the KEGG database (https://www.genome.jp/kegg/pathway.html, accessed on 31 January 2024), the HMDB database (https://hmdb.ca/metabolites, accessed on 31 January 2024), and the LIPIDMaps database (http://www.lipidmaps.org/, accessed on 31 January 2024) were employed to ensure comprehensive and accurate annotations of the metabolites. DAMs were selected using a threshold of variable importance in projection (VIP) ≥ 1, a fold change of 2, and *p* ≤ 0.05. The method chosen for calculating the *p*-value is ANOVA (https://cloud.metware.cn, accessed on 17 August 2024). Finally, pathway and other functional analyses were conducted to uncover the biological significance of the metabolites.

### 4.5. Gene Expression Analyses via qRT-PCR

In order to ensure the precision and dependability of the transcriptome data, a selection of 20 genes was randomly chosen for validation through real-time quantitative PCR (qRT-PCR). *GbUBQ7* was identified as the reference gene candidate based on the sequencing data from the transcriptome. The design of specific primers was accomplished using the Primer-BLAST tool available on the NCBI website, with primer sequences detailed in Table S4. The RNA samples used were the same as those returned from the transcriptome sequencing conducted by iGeneTech Co., Ltd., Seoul, Republic of Korea cDNA was synthesized from RNA through reverse transcription using the HiScript III RT SuperMix specifically designed for quantitative PCR, which includes a gDNA wiper to eliminate genomic DNA contamination. Subsequently, qRT-PCR assays were conducted with a ChamQ Universal SYBR qPCR Master Mix (Vazyme, Nanjing, China). The quantification of gene expression levels was carried out utilizing the 2^−ΔΔCt^ approach [75]. Three independent biological replicates along with three technical replicates were set up for both the control and experimental groups to ensure reproducibility and data reliability.

## 5. Conclusions

In this study, to identify key DEGs and DAMs related to resistance to VW in *G. hirsutum* and *G. barbadense*, and cultivate new disease-resistant varieties, RNA-seq and metabolomic analyses were conducted on root tissues of the resistant material Hai7124 and the susceptible material TM-1 at the early stages (0, 1, and 2 DAI) of V991 infection. The analysis of DEGs and DAMs using KEGG enrichment demonstrated that nine pathways were co-enriched, including glutathione metabolism, propionate metabolism, and pyrimidine metabolism. Through joint analysis, the glutathione metabolism pathway was identified as potentially significantly contributing to the early response of cotton to VW infection. A glutathione metabolism pathway map was constructed, revealing a significant positive correlation between the metabolite putrescine and DEGs within GSTs. Based on the comprehensive analysis, it is suggested that the DEG *GH_D11G2329* (*GSTF8*) could play a role in cotton response to VW infection. These results provided abundant information for screening the crucial VW-resistant genes and metabolites, thereby establishing a strong basis for further studying the molecular mechanism behind plant defense against VW infection.

## Figures and Tables

**Figure 2:**
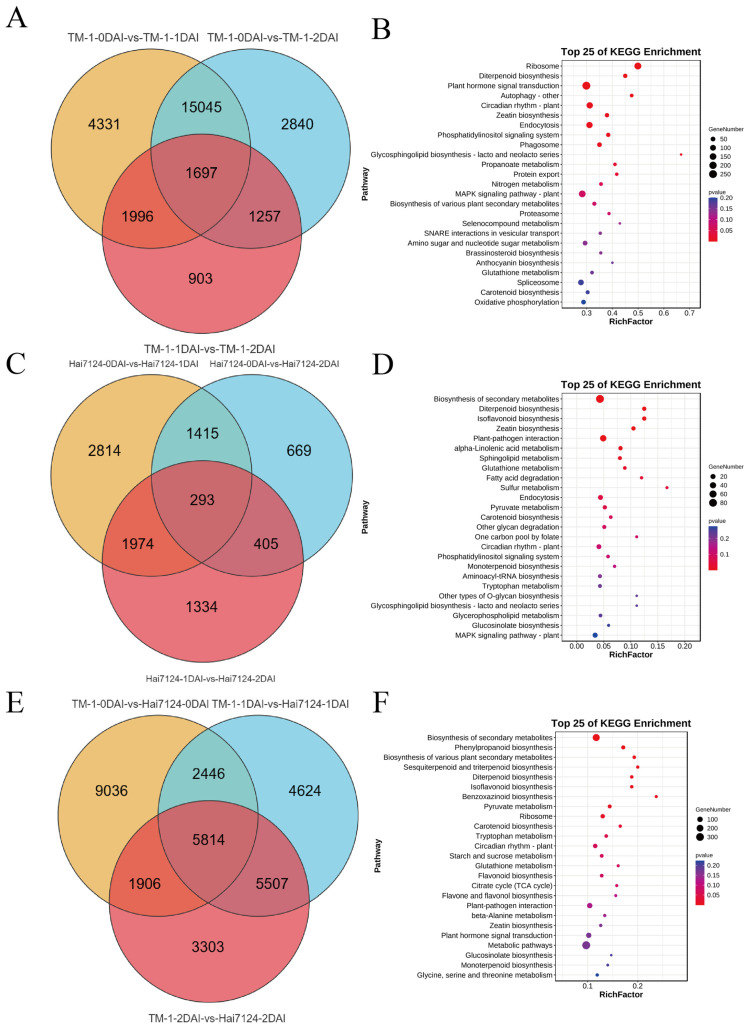
Analysis of Functional Enrichment for Genes with Differential Expression (**A**) Venn diagram of 1697 DEGs across three different infection stages in TM-1. (**B**) KEGG enrichment analysis results of in common DEGs in TM-1. (**C**) Venn diagram of 293 DEGs across three different infection stages in Hai7124. (**D**) KEGG enrichment analysis results of in common DEGs in Hai7124. (**E**) Venn diagram of 5814 DEGs at the same infection stage across different materials. (**F**) Enrichment analysis results in common DEGs at the same infection stage across different material.

## Data Availability

The original contributions presented in the study are included in the article/Supplementary Materials, and further inquiries can be directed to the corresponding authors.

## Supplementary Materials

The supporting information can be downloaded at: https://www.mdpi.com/article/10.3390/ijms26010028/s1.
