# Supplementary material for: Integrated Transcriptomic and Metabolomic Analysis of G. hirsutum and G. barbadense Responses to Verticillium Wilt Infection
Source: Int J Mol Sci. 2024 Dec 24;26(1):28. doi: 10.3390/ijms26010028 (PMC11720156; doi:10.3390/ijms26010028)
Supplement: Supplementary file 1 [file ijms-26-00028-s001.zip › ijms-3324562-supplementary.pdf]

Table S1 Overview of sequencing data and comparison results with reference genomes

Sample	Clean reads(M)	Clean bases(G)	Q30(%)	GC(%)	Total mapped(%)
T0-1	48.25	7.24	92.56	45.52	96.63%
T0-2	48.11	7.22	92.75	44.75	96.32%
T0-3	48.30	7.24	91.95	45.01	96.28%
T24-1	48.41	6.94	94.19	43.68	95.80%
T24-2	48.65	6.97	93.91	43.67	96.95%
T24-3	49.15	7.03	94.21	43.76	96.98%
T48-1	48.96	7.01	93.87	43.74	96.97%
T48-2	48.34	6.95	93.6	44.6	95.04%
T48-3	48.27	6.97	93.85	43.73	97.27%
H70-1	40.80	6.11	95.64	43.43	95.13%
H70-2	41.54	6.20	96.67	44.96	95.84%
H70-3	40.88	6.10	97.08	45.14	96.29%
H724-1	41.03	6.09	97.51	45.61	94.95%
H724-2	41.32	6.17	96.83	45.02	96.05%
H724-3	40.84	6.11	96.66	45.03	94.67%
H748-1	42.04	6.27	97.26	45.09	96.66%
H748-2	38.12	5.70	95.94	43.58	94.04%
H748-3	39.87	5.97	96.68	44.44	96.00%

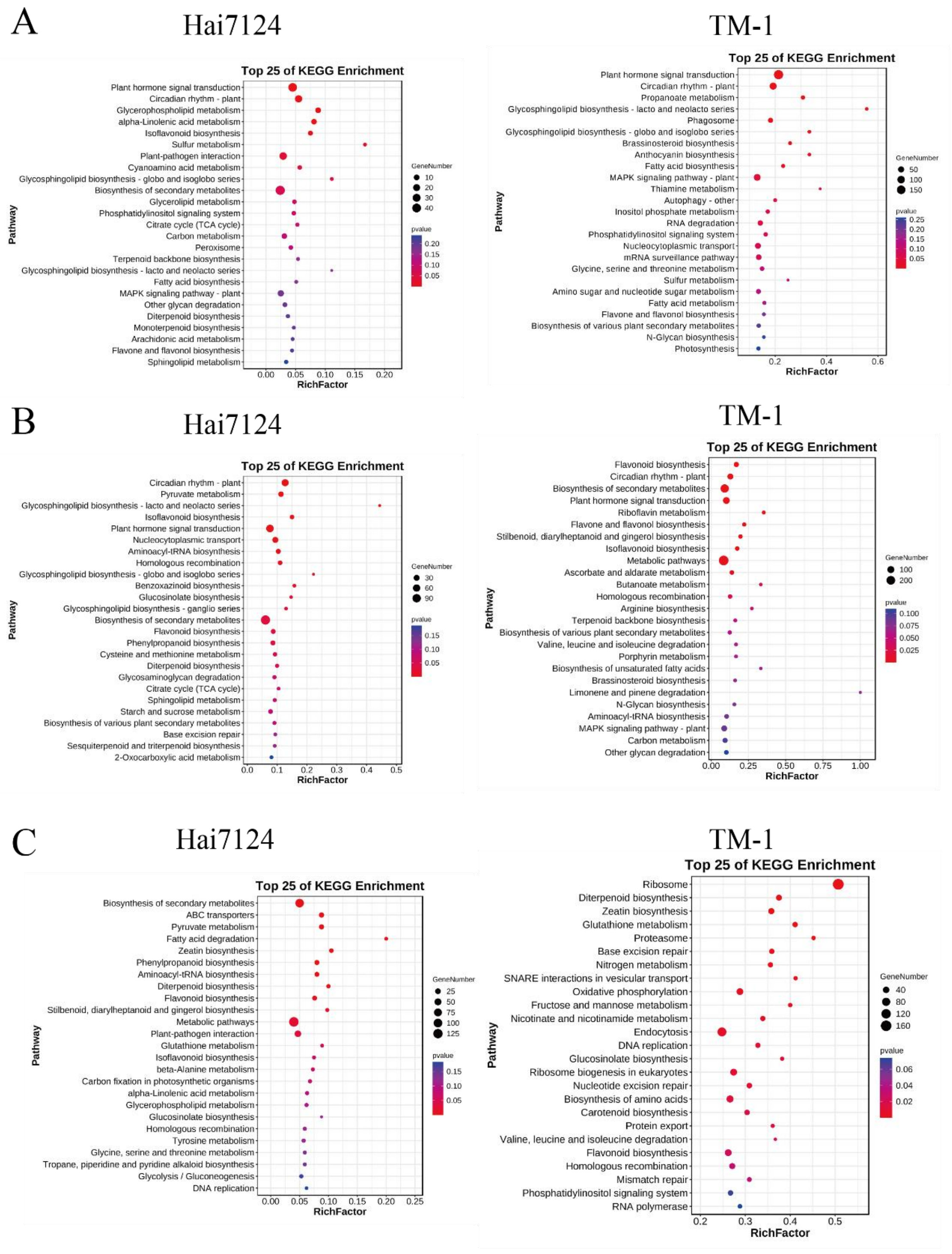

Figure S1 KEGG Enrichment Analysis of Expression Patterns.

(A) KEGG enrichment analysis for Expression Profile 0 in Hai712 and TM-1. (B) KEGG enrichment analysis for Expression Profile 1 in Hai712 and TM-1. (C) KEGG enrichment analysis for Expression Profile 7 in Hai7124 and TM-1.

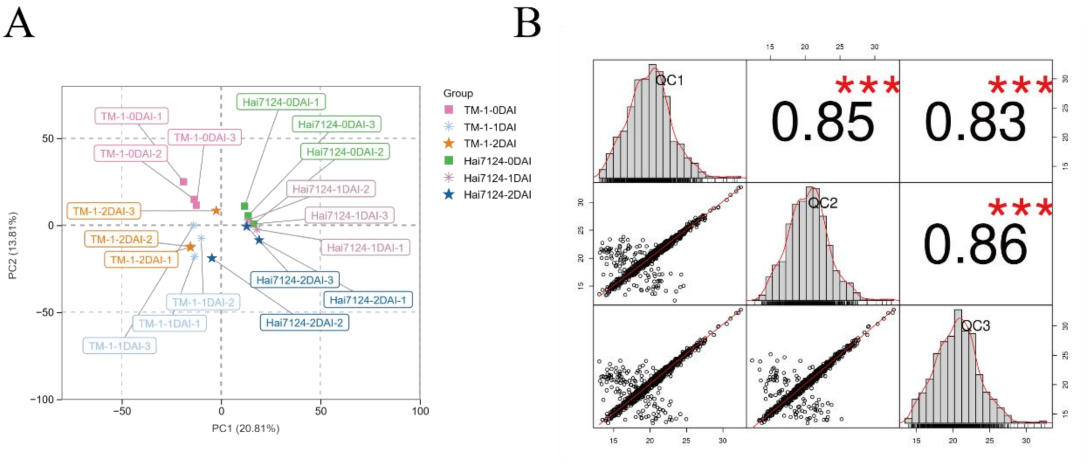

Figure S2 Quality Control Analysis of Metabolomic Sequencing Data.
(A) Principal Component Analysis (PCA). (B) Quality Control (QC) Correlation Analysis.

Table S2 Metabolite species that continuously increase with the duration of *Verticillium dahliae* infection

Description	tax_Class
11Z-Octadecenylcarnitine	Others
2-Amino-9,10-epoxy-8-oxodecanoic acid	Others
Methyl palmitate	Fatty Acyls
ACar 20:5	Others
Ceramide (d18:1/20:0)	Others
Butanoic acid	Fatty Acyls
(5-L-Glutamyl)-L-Amino Acid	Others
2-methylhippuric acid	Benzene and substituted derivatives
Lignoceric Acid	Fatty Acyls
Dihydrothymine	Diazines
2-(3,5-dimethyl-1H-pyrazol-4-yl)-5-methoxybenzoic acid	Others
(2S)-2-(2-hydroxypropan-2-yl)-2H,3H,7H-furo[3,2-g]chromen-7-one	Others
6-phenyl-1,2,3,4-tetrahydro-2,5-benzodiazocin-1-one	Others
N-Caffeoyl Putrescine	Others
3-methyl-5-oxo-5-(4-toluidino)pentanoic acid	Others
(R)-3-Hydroxy myristic acid	Others
1,5-Anhydro-D-glucitol	Organooxygen compounds
N-(3-Oxododecanoyl)homoserine lactone	Others
Indirubin	Indoles and derivatives
2-(2-oxo-2-{[2-(2-oxo-1-imidazolidinyl)ethyl]amino}ethoxy)acetic acid	Others
Linustatin	Organooxygen compounds
Estrone sulfate	Steroids and steroid derivatives
PC(14:1(9Z)/P-18:1(11Z))	Glycerophospholipids
PC (16:1/16:1)	Others

Description	tax_Class
ACar 18:0	Others
PC(14:0/P-16:0)	Glycerophospholipids
Ceramide (d18:1/16:0)	Sphingolipids
PC (18:1e/14:1)	Others
PC(14:1(9Z)/18:2(9Z,12Z))	Glycerophospholipids
SM(d18:0/16:1(9Z)(OH))	Sphingolipids
N-cyclohexyl-1-methyl-5-(1H-pyrrol-1-yl)-1H-pyrazole-4-carboxamide	Others
L-Cysteine-glutathione gisulfide	Others
Oxoadipic Acid	Keto acids and derivatives
GNH	Others
D-Glucoronic Acid	Others
Description	tax_Class
allopurinol	Pyrazolopyrimidines
10-Hydroxydecanoic acid	Hydroxy acids and derivatives
N-(3-Oxododecanoyl)homoserine lactone	Others
Mevalonic acid	Fatty Acyls
LysoPC(18:1(11Z))	Glycerophospholipids
Protectin D1	Fatty Acyls
2-[(3S)-1-(1-Methyl-4-piperidinyl)-3-pyrrolidinyl]-1H-benzimidazole	Others
Prostaglandin E2	Fatty Acyls
Prostaglandin K2	Others
Dehydroepiandrosterone (DHEA)	Others
N-[4-(tert-butyl)phenyl]-N-cyclohexyl-N-methylurea	Others
SM (d14:0/16:2)	Others
Pyridoxamine	Pyridines and derivatives

Description	tax_Class
Celastrol	Prenol lipids
Cinchophen	Quinolines and derivatives

Table S3 Correlation analysis of DEGs and DAMs in the glutathione metabolism signaling pathway

Genes	Metabolite	Correlation	P-Value
GH_A11G2109	Putrescine	0.814709019	0.000038
GH_D04G0634	Putrescine	0.86028555	0.000005
GH_D11G2329	Putrescine	0.861562289	0.000004
GH_D13G1106	Putrescine	0.819207153	0.000032

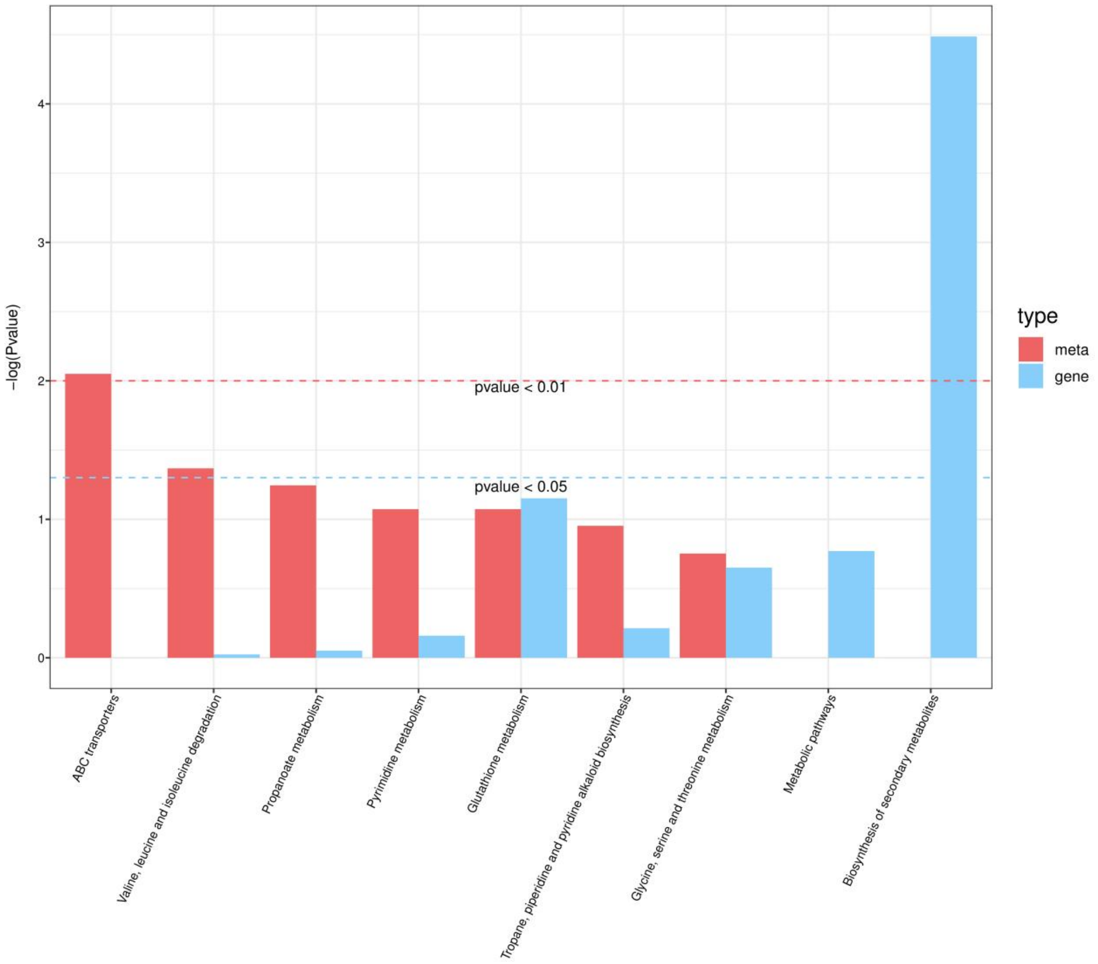

Figure S3 Conjoint enrichment analysis of DAMs and DEGs from transcriptomic and metabolome sequencing

Table S4 Primer design for 20 randomly selected DEGs

ID	Primer	5' to 3'
GH_D02G0428	1-F	CAAAGAAGGAGAAGCCACCG
	1-R	TGGTTTCTTGGGAGACTCATGT
GH_A05G0151	2-F	GGCAGCACTTCTTGTTGTTT
	2-R	CACAAGAATATCCCCAACCACG
GH_D10G1713	3-F	AATACACAGTGGACCTGGCG
	3-R	TGGGTGCTTTATCGGTAGGG
GH_A01G2417	4-F	TTTGCCGAAAGACTTGTGCG
	4-R	TGCCGACGGAGTTTCTATCG
GH_D01G2494	5-F	CCGCCGGTGAAGGTGTATTT
	5-R	GCAGAAGCAACCTAAGCACA
GH_D03G1575	6-F	TCATCAGCACCAAGTGACCC
	6-R	CACAGTGATGCCAATGTGCC
GH_D12G2460	7-F	TAGGCATGAGTTTGCCCCTG
	7-R	ACCAGTACAGTTCCCGTCCT
GH_A08G2617	8-F	TACGGACGGTGAGAAAACGG
	8-R	GTGCCAAAACATCGGCACAT
GH_A07G0848	9-F	CAAATTCGACAAAAACGGCG
	9-R	TGCTTCACCATCGTCGTCAT
GH_A09G0838	10-F	AGCTTACTGGCCGAGTGTTC
	10-R	CGATCGAACCGCGTTGAATA
GH_D05G3216	11-F	GACACCCATATTGGCAGGGA
	11-R	GAGTGCCTTTGGGGATGTCA
GH_A07G1847	12-F	GGCACTCTTCAGGTGTTTGC
	12-R	TCCCAATGGTCATCGGGTTTA
GH_A13G0381	13-F	CCACACTGGTCAGGGCTTTA
	13-R	CGCTGTGAGGGAATGTGGTA
GH_D07G1830	14-F	TGGCTTCGACATCCTTCATCC
	14-R	CCATTGATGCTGTGAGAGCG
GH_D05G1803	15-F	GTGTGCACTGGTTATGGCTG
	15-R	CCAATGGTCTCAGGATTGCCA
GH_A11G3220	16-F	GCCAATGCACGAAGGGAAAC
	16-R	TGCACTTGCCATCGTTCTCA
GH_D01G0012	17-F	ACGACTGCAGAACGGGAAAT
	17-R	TACTTCAGGGCAACGGAACC
GH_D10G1954	18-F	TTTGAGGCAGCTGTAGGTGG
	18-R	AGTTGCAAGCATCGGGGTTA
GH_D08G2346	19-F	TCATTTTTCGCGGACGACGG
	19-R	TCTCCGATGCTTCGGTCATT
GH_A12G1536	20-F	ACATGACGAGCTTGTGAGCA
	20-R	AGCGGAAGAATTGAGGTCGT

ID	Primer	5' to 3'
GH_A03G0218	21-F	TGACCGGTGACGAAAGCTAC
	21-R	CCGCTTTCCTGCCAATTCAC
GH_A01G0012	22-F	GATCTTGCCGGTCGTGATCT
	22-R	TTGACCATCGGGCAATTCGT
GH_D02G2251	23-F	TGTTCATAGGCCGGGATTGG
	23-R	CCAATACCCTTCCCACGCTT
GH_D01G2266	24-F	GCAACTAACGGCAGAGCAAG
	24-R	AACATCTCCCATGCCCACAA
GH_A01G1015	25-F	CGGCAAAGCTGGTGGAGA
	25-R	TTGGGACCCTCAGCCTGT
GH_A08G2342	26-F	TCGAAAGTTCCGGTTGGGAG
	26-R	CGGTGGGTAGTGCTTCTTGT
GH_D08G2368	27-F	TTGATGGTGGCAGTTGATGC
	27-R	TTGAAGAGCGCCGTAGCTTA
GH_D13G0077	28-F	CGCCGTGCTCCTCTTATTCT
	28-R	GATTCCGACGTGGGGTTTCT
GH_D13G1213	29-F	CCAGGTCATGGTCGGTTTCA
	29-R	GTCGGGGTTCCCCAAATCAT
GH_D08G2611	30-F	TGCCGATGTTTTGGCACTTG
	30-R	GTGTGAACACCGGACAAAGC
GH_D12G1539	31-F	GAGACACGACGAGCTTGTGA
	31-R	AGCGGAAGAATTGAGGTCGT
GH_A13G2325	32-F	TCGATTTTCTCTAGCAGCCTTC
	32-R	GAAGGCAAAAAGGAAGTAGCC
GH_D10G1351	33-F	TGGCCATTTTGAGCCATGTTG
	33-R	ACTTCGCCTTCTCGTACACC
GH_D05G1190	34-F	GGCGCCTCTGAATCTGAAGT
	34-R	TGCTGCCTTTGTTTCTCCTCT
GH_D12G1964	35-F	GACTATCCCTCCCTAATGGAATTG
	35-R	AAAATGGCACCCTTCACAAC
GH_D13G1634	36-F	TGGCTAACAAGCTTGGAGGG
	36-R	GCACCATCGCCATCGAAATC
GH_A03G1397	37-F	AGCTCCTCCAACGCCTACTA
	37-R	TTGGCCAAAAGTCACGCAAG
GH_A03G1411	38-F	GTCTTCAGCTCTGACGGGTA
	38-R	CGACTTTCCCCCATTCACCT
GH_A03G0460	39-F	GCGCCAATCACTAGCAGAAG
	39-R	AGAAAATGGTTCGGGGCAGA
GH_D05G1740	40-F	TGGGTATTGACACCCCTGGA
	40-R	ATGTGTGGAACATGGTCGGG
GHUBQ7	41-F	GAAGGCATTCCACCTGACCAAC
	41-R	CTTGACCTTCTTCTTCTTGTGCTTG
